# The shifting epidemiology and serotype distribution of invasive pneumococcal disease in Ontario, Canada, 2007-2017

**DOI:** 10.1371/journal.pone.0226353

**Published:** 2019-12-13

**Authors:** Shinthuja Wijayasri, Kelty Hillier, Gillian H. Lim, Tara M. Harris, Sarah E. Wilson, Shelley L. Deeks

**Affiliations:** 1 Communicable Diseases, Emergency Preparedness and Response, Public Health Ontario, Toronto, Ontario, Canada; 2 Dalla Lana School of Public Health, University of Toronto, Toronto, Ontario, Canada; Universidad Nacional de la Plata, ARGENTINA

## Abstract

**Background:**

Ontario, Canada introduced a publicly-funded 13-valent pneumococcal conjugate vaccine (PCV13) for infants in 2010, replacing the 10-valent (PCV10, 2009–2010) and the 7-valent (PCV7, 2005–2009) conjugate vaccine programs; a 23-valent pneumococcal polysaccharide vaccine (PPV23) has been available for older adults since 1996. We examined the epidemiology and serotype distribution of invasive pneumococcal disease (IPD) in Ontario in the context of provincial immunization programs.

**Methods:**

We included confirmed IPD cases reported in Ontario between 2007 and 2017. We grouped serotypes according to Ontario’s current immunization program (PCV13, PPV23, and non-vaccine-preventable) and calculated incidence rates (per 100,000 population) using population data.

**Results:**

Between 2007 and 2017, annual incidence of IPD in Ontario ranged between 7.3 and 9.7/100,000 per year. Measures of illness severity were high throughout the period of surveillance. After PCV13 program implementation in 2010, incidence due to PCV13 serotypes decreased significantly across all age groups, with the greatest reductions in children <5 years and adults ≥65 years. Conversely, incidence due to PPV23 unique serotypes increased significantly between 2007 and 2017, with the greatest increases observed in adults 50–64 years (1.4 to 3.5/100,000) and ≥65 years (2.3 to 7.2/100,000). Similar increases were observed in incidence due to non-vaccine-preventable serotypes among all age groups, except infants <1 year. Within specific serotypes, incidence due to serotypes 3 (0.42 to 0.98/100,000) and 22F (0.31 to 0.72/100,000) increased significantly between 2007 and 2017, while incidence due to serotypes 19A and 7F decreased significantly during the PCV13 period (2010–2017).

**Conclusions:**

Eight years after PCV13 implementation in Ontario, our data suggest both direct and indirect effects on serotype-specific incidence in young children and older adults. However, overall provincial rates have remained unchanged, and IPD continues to be a severe burden on the population. The rising incidence of IPD due to PPV23 unique and non-vaccine-preventable serotypes, and the growing burden of serotypes 3 and 22F, require further study.

## Introduction

Invasive pneumococcal disease (IPD), caused by *Streptococcus pneumoniae*, is associated with severe morbidity and mortality, particularly in young children and the elderly [1,2]. It is an important cause of severe illness, including meningitis, bacteremia and bacteremic pneumonia [1]. There are currently 97 serotypes of *S*. *pneumoniae* recognized worldwide, a subset of which cause the majority of disease in Canada [1,3]. The introduction of publicly-funded pneumococcal conjugate vaccines has resulted in drastic declines in vaccine-specific serotypes among children in Canada [4,5]. However, incidence of IPD among older adults have remained generally unchanged [6]. IPD continues to represent a substantial disease burden in Canada, with incidence ranging between 8.9 and 9.9 per 100,000 population between 2003 and 2015 [6].

In 2010, Ontario, Canada (population approximately 14 million) introduced a publicly-funded three-dose 13-valent pneumococcal conjugate vaccine (PCV13) program for infants at 2, 4 and 12 months, replacing a four-dose 10-valent conjugate vaccine program (PCV10, in effect from 2009 to 2010) and a four-dose 7-valent conjugate vaccine program (PCV7, in effect from 2005 to 2009) [7]. A single dose 23-valent polysaccharide vaccine (PPV23) program for adults 65 years and older and those considered high risk for IPD has been in place since 1996 and a high risk PCV13 immunization program, targeting adults over age 50 years of age with medical risk factors, was implemented in 2014 [7]. While reductions in incidence and shifts towards an older age distribution suggest that Ontario’s immunization program has been effective in reducing the burden of IPD in children, previous data also suggest shifts in circulating serotypes and serotype replacement following the introduction of pneumococcal conjugate vaccines [8,9].

With the PCV13 program now in place for eight years, there is an opportunity to assess the long term effect of the program on serotype distribution. We examined the epidemiology of IPD and the distribution of circulating *S*. *pneumoniae* serotypes in Ontario between 2007 and 2017, in the context of the provincial immunization programs.

## Methods

### Case definition and surveillance data

Under the *Health Protection and Promotion Act*, all public health units (PHUs) in Ontario are responsible for case management and reporting of IPD [10]. PHUs classify cases using provincial surveillance case definitions, and report confirmed cases using Ontario’s integrated Public Health Information System (iPHIS). Confirmed IPD is defined as clinical evidence of invasive disease and the isolation of *S*. *pneumoniae* or detection of *S*. *pneumoniae* DNA from a normally sterile site (e.g. blood, cerebrospinal fluid, excluding the middle ear) [11]. PHUs report information on demographics, clinical illness, laboratory information, immunization history, hospitalizations and deaths.

In Ontario, most diagnostic laboratories forward isolates of *S*. *pneumoniae* from sterile sites to the National Microbiology Laboratory (NML) via Public Health Ontario (PHO) Laboratory for serotyping, and results are reported to the PHUs and entered into iPHIS. The exception is the Toronto Invasive Bacterial Diseases Network (TIBDN), which covers a surveillance population of approximately 4 million [12]. Hospital sites participating in TIBDN forward isolates from sterile sites to the TIBDN laboratory for *S*. *pneumoniae* identification. These isolates are serotyped at the TIBDN laboratory, the Alberta Provincial Public Health Laboratory or NML [13]. Results from TIBDN are entered into iPHIS centrally by PHO. IPD cases and serotyping results entered into iPHIS are routinely validated by PHO to ensure that they meet the appropriate case definitions. Given that routine reporting of serotype data began in 2007 [14], we extracted details from iPHIS on all confirmed IPD cases occurring between January 1, 2007 and December 31, 2017 for this analysis. There was a change to laboratory methods and the confirmed case definition during this study period–nucleic acid testing (NAT), a more sensitive diagnostic methodology, was added to the case definition in 2009 [7].

### Serotype groupings

We grouped IPD cases by associated vaccine type. Serotypes covered by the conjugate vaccines (PCV7, PCV10 and PCV13) were designated as ‘PCV13’ (serotypes 1, 3, 4, 5, 6A, 6B, 7F, 9V, 14, 18C, 19A, 19F and 23F) [15]. Serotypes covered by the polysaccharide vaccine (PPV23) but not the conjugate vaccines (serotypes 2, 8, 9N, 10A, 11A, 12F, 15B, 17F, 20, 22F and 33F) were designated as ‘Unique PPV23’; there is one serotype in PCV13 that is not in PPV23 (6A) [15]. All serotypes that are not currently preventable by pneumococcal vaccines in Canada were designated as ‘non-vaccine-preventable’.

### Analyses

We conducted descriptive analyses using SAS Enterprise Guide 7.1 (SAS Institute Inc., Cary, NC, USA). We used case-level data from iPHIS to describe demographic characteristics and clinical outcomes of IPD. We defined ‘infants’ as persons under one year of age, and ‘older adults’ as persons 65 years of age and older. We evaluated changes in median age over the study period using the Mann-Whitney *U* test. We grouped Ontario’s 35 PHUs into seven regions for geographic analyses. We calculated incidence rates per 100,000 population using population estimates (2007–2016) and projections (2017) obtained from Statistics Canada [16]. Due to over-dispersion in the data, we used negative binomial regression to assess trends in incidence rates over time. We considered *p*-values less than 0.05 as statistically significant. When assessing immunization status, we included only documented doses of a pneumococcal vaccine product administered at least 14 days prior to disease onset.

### Ethics

This manuscript reports on routine surveillance activities carried out under provincial legislation (*Ontario Agency for Health Protection and Promotion Act*, *SO 2007*, *c 10)* and not research, and therefore research ethics committee approval was not required.

## Results

### Overall

Between 2007 and 2017, 12,377 confirmed cases of IPD were reported in Ontario, representing an annualized incidence rate of 8.4 per 100,000 population. The annual incidence rate ranged between 7.3 and 9.7 per 100,000 population, however no statistically significant trend was observed over the 11-year period (*p* = 0.20). We observed geographical variations in incidence within the province, with the highest annualized incidence rates in the North West and North East regions (17.5 and 10.3 per 100,000 population, respectively), and the lowest in the Toronto and Central East regions (7.8 and 6.9 per 100,000 population, respectively). The median age of cases was 61 years (range: less than one day-104 years) and males had a higher rate of disease than females (**Table 1**).

**Table 1:** Characteristics of confirmed invasive pneumococcal disease cases reported in Ontario, Canada, 2007–2017 (N = 12,377).

Characteristics	% of Cases (n)		Annualized Rate ^a^ (per 100,000 population)
**ONTARIO (OVERALL)**	100	(12,377)	8.4
**Gender** ^b^			
** **Male	53.8	(6655)	9.2
** **Female	46.2	(5714)	7.6
**Region**			
** **Central East	24.0	(2974)	6.9
** **Central West	21.8	(2697)	9.5
** **Eastern	13.9	(1724)	8.9
** **North East	5.2	(641)	10.3
** **North West	3.7	(457)	17.5
** **South West	12.3	(1517)	8.6
** **Toronto	19.1	(2367)	7.8
**Age (years)**			
** **<1 year	1.8	(223)	14.3
** **1–4 years	6.2	(773)	12.2
** **5–49 years	22.4	(2777)	3.2
** **50–64 years	26.6	(3288)	11.0
** **65+ years	43.0	(5316)	24.2
**Hospitalizations, by age group** ^c^			
** **<1 year	75.3	(168/223)	10.7
** **1–4 years	71.5	(553/773)	8.8
** **5–49 years	69.9	(1940/2777)	2.2
** **50–64 years	73.0	(2399/3288)	8.0
** **65+ years	76.9	(4088/5316)	18.6
** **Overall (Ontario)	73.9	(9148/12,377)	6.2
**Deaths, by age group** ^**c**^			
** **<1 year	4.0	(9/223)	0.6
** **1–4 years	1.6	(12/773)	0.2
** **5–49 years	5.7	(159/2777)	0.2
** **50–64 years	10.9	(359/3288)	1.2
** **65+ years	17.2	(917/5316)	4.2
** **Overall (Ontario)	11.8	(1456/12,377)	1.0

^a^ Rates are specific to each subgroup indicated.

^b^ Cases with unknown gender were excluded (n = 8).

^c^ % is proportion of all cases (n = 12,377) unless otherwise indicated; for hospitalizations and deaths, % refers to the proportion of cases within each age group indicated.

Of the 12,377 cases, hospitalization was reported for 73.9%, and was high across all ages (at least 69.9% hospitalized in each age group). Hospitalization was highest in older adults (76.9%) and infants (75.3%). The provincial case fatality ratio (CFR) during this period was 11.8%. CFR varied greatly across age groups; the CFR in those 50–64 years (10.9%) and older adults (17.2%) were respectively 2.7 and 4.3 times higher than the 4.0% CFR of infants. The annualized death rate in older adults was seven times that of infants (**Table 1**). Immunization status was known for only 37.6% of cases (4654/12,377), of whom 67.9% (3160/4654) were reported as having not received any pneumococcal vaccine product.

### Age group trends

**Fig 1:**
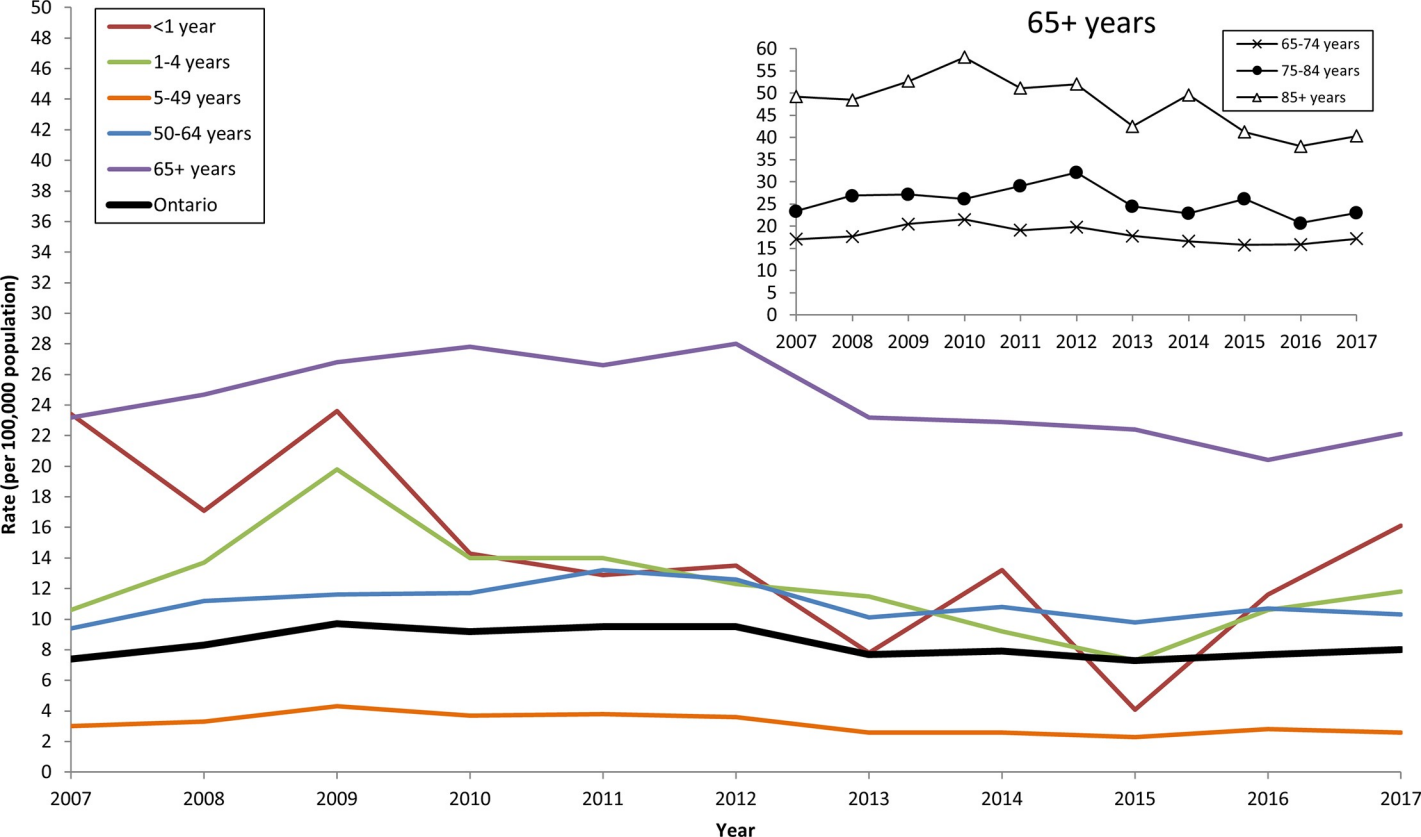
Incidence rates (per 100,000 population) of invasive pneumococcal disease, by age group and year, in Ontario, Canada, 2007–2017.

The annual median age of cases increased from 60.4 years in 2007 (IQR: 41.1–75.1 years) to 63.5 years in 2017 (IQR: 48.0–75.9 years) (*p* = 0.004). Significant reductions in age-specific incidence were observed in infants (*p* = 0.02) and children 1–4 years old (*p* = 0.03), reaching a low of 4.1 and 7.3 per 100,000 population (respectively) in 2015 (**Fig 1**). However, rates in these age groups have increased since 2015, reaching 16.1 (infants) and 11.8 (1–4 years olds) per 100,000 population in 2017. A significant decrease in incidence was also observed in older adults between 2007 and 2017 (*p* = 0.01). When stratifying this age group further, a significant decrease was observed among those 65–74 years (*p* = 0.03) and those 85 years and older (*p* < .001); no statistically significant trend was observed in those 75–84 years old (*p* = 0.15) (**Fig 1, inset**). There were also no significant trends in the annual incidence of IPD in those aged 50–64 years old over the study period (*p* = 0.44). Incidence in the 5–49 year age group have been low and stable (ranging from 2.3 to 4.3 per 100,000 population per year), with an annualized rate of 3.2 per 100,000 population during the study period (**Fig 1**, **Table 1**).

### Serotype distribution by associated vaccine type

**Fig 2:**
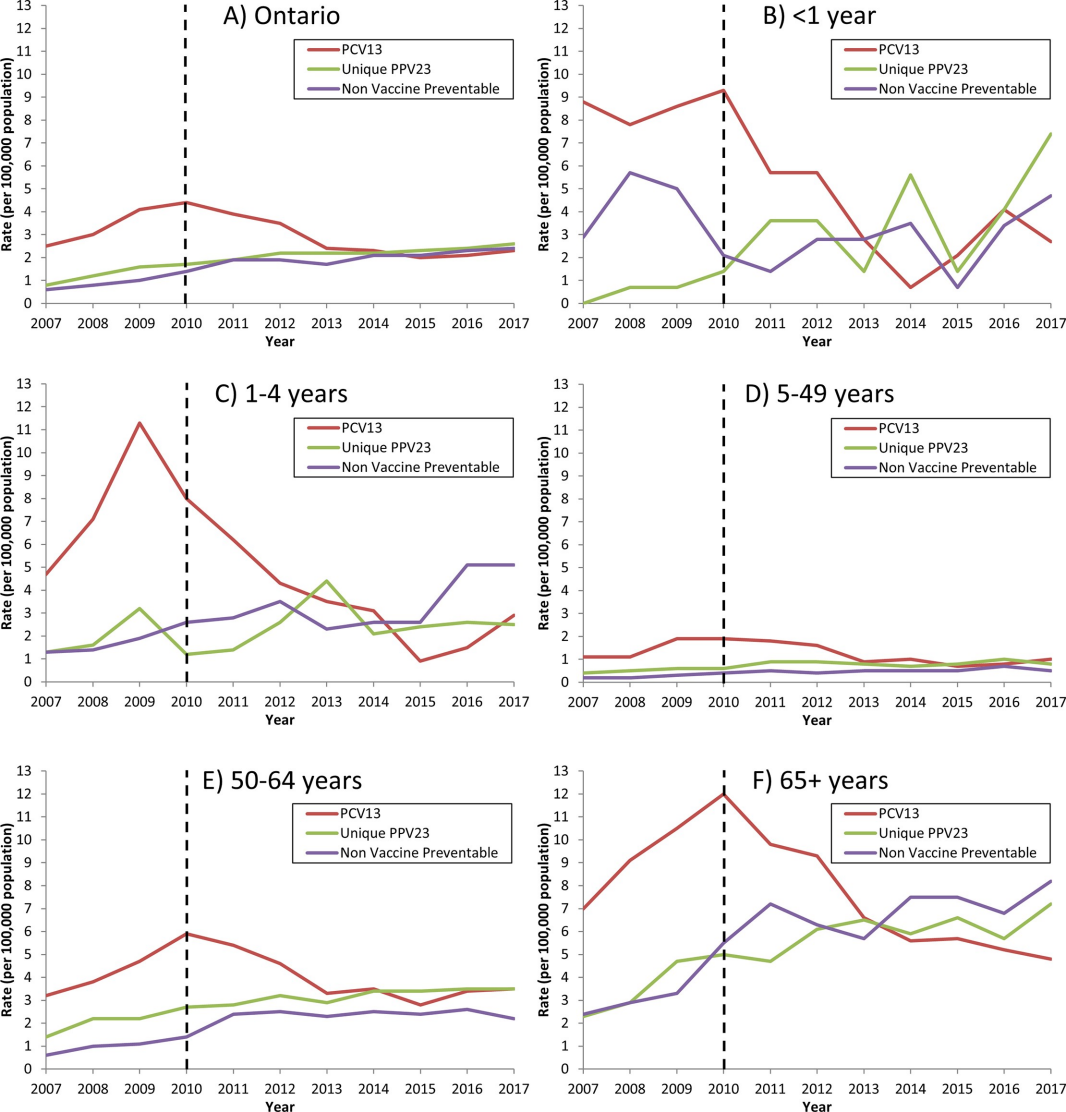
Incidence rates (per 100,000 population) of invasive pneumococcal disease, by year, associated vaccine type^a^ and age group, in Ontario, Canada, 2007-2017^b^. ^a^ Includes only cases with available serotype information (9725/12,377, 78.6%); cases with non-typeable serotypes were included in the non-vaccine-preventable category (n = 25). ^b^ Dotted line indicates implementation of pediatric PCV13 immunization program in Ontario (2010). Abbreviations: PCV13: 13-valent pneumococcal conjugate vaccine; PPV23: 23-valent pneumococcal polysaccharide vaccine.

Serotype information was available for 78.6% of cases (9725/12,377). Of these, 44.7% were due to serotypes covered by the PCV13 vaccine, 29.5% due to serotypes unique to PPV23 and 25.8% due to non-vaccine-preventable serotypes. Overall provincial incidence due to PCV13 serotypes decreased between 2007 and 2017 (*p* = 0.007). Incidence peaked at 4.4 per 100,000 population in 2010, the year the PCV13 program was introduced, and decreased to 2.0 per 100,000 population in 2015, representing a 54.6% reduction within six years; incidence has since increased to 2.3 per 100,000 population in 2017 (**Fig 2A**). After stratifying by age group, we observed significant decreasing trends in incidence due to PCV13 serotypes among all age groups (*p* < .001) during the PCV13 program period (2010 to 2017). We saw the largest reductions in children–specifically, infants between 2010 and 2014 (9.3 to 0.7 per 100,000 population, 92.5% reduction) and children 1–4 years old between 2009 and 2015 (11.3 to 0.9 per 100,000 population, 92.0% reduction). However, incidence due to PCV13 serotypes in both these age groups has risen in recent years (**Fig 2B**, **Fig 2C**). In adults aged 50–64 years, incidence due to PCV13 serotypes decreased by 44.1% in the first three years after PCV13 program implementation (from 5.9 to 3.3 per 100,000 population between 2010 and 2013); annual incidence has since stabilized in this age group (**Fig 2E**). Incidence due to PCV13 serotypes in older adults has declined significantly during the PCV13 program period, from a peak of 12.0 per 100,000 population in 2010 to 4.8 per 100,000 population in 2017 (*p* < .001), representing a 60.0% reduction in eight years (**Fig 2F**).

We noted significantly increased overall incidence due to serotypes unique to PPV23 (*p* < .001) and non-vaccine-preventable serotypes (*p* < .001) over the study period (**Fig 2A**). When stratified by age group, these increases disproportionately affected older age groups. We observed variability in annual incidence due to serotypes unique to PPV23 in children under five years, with a significant increasing trend in infants (*p* = 0.002), but no significant trend in children aged 1–4 years (*p* = 0.13) (**Fig 2B**, **Fig 2C**). Increases also occurred in adults aged 50–64 years, from 1.4 to 3.5 per 100,000 population between 2007 and 2017 (*p* < .001) (**Fig 2E**). However older adults had the greatest increase in incidence due to serotypes unique to PPV23, with incidence more than tripling between 2007 and 2017 (from 2.3 to 7.2 per 100,000 population, *p* < .001) (**Fig 2F**). We noted similar age-specific trends for non-vaccine-preventable serotypes; significantly increasing trends were observed in all age groups (*p* < .001) except infants (p = 0.58). In particular, increases in incidence due to non-vaccine-preventable serotypes was greatest in older adults (from 2.4 to 8.2 per 100,000 population between 2007 and 2017, *p* < .001) (**Fig 2F**).

### Distribution of specific serotypes

**Fig 3:**
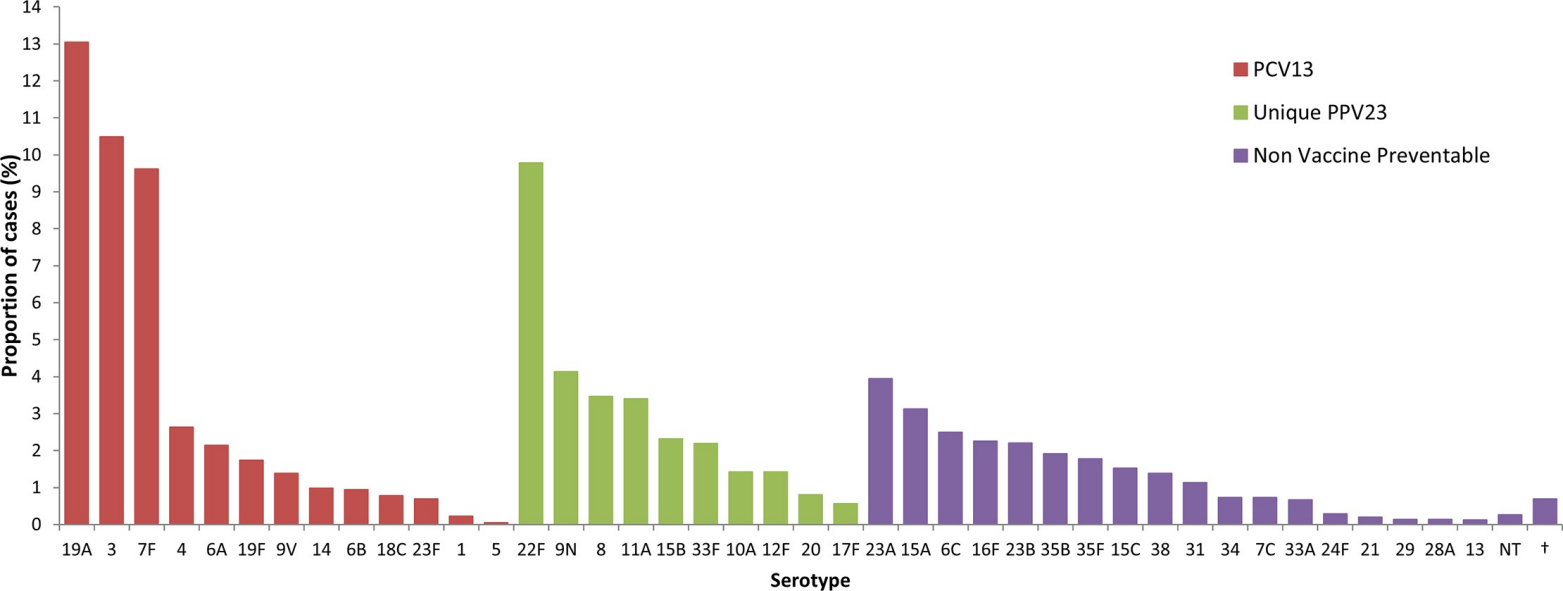
Serotype distribution of invasive pneumococcal disease cases^a^, by associated vaccine type, in Ontario, Canada, 2007–2017. ^a^ % are proportion of cases with available serotype information (n = 9725). † Includes 28 other non-vaccine-preventable serotypes that individually accounted for <0.1% of cases with available serotype information. Abbreviations: PCV13: 13-valent pneumococcal conjugate vaccine; PPV23: 23-valent pneumococcal polysaccharide vaccine; NT: non-typeable.

**Fig 4:**
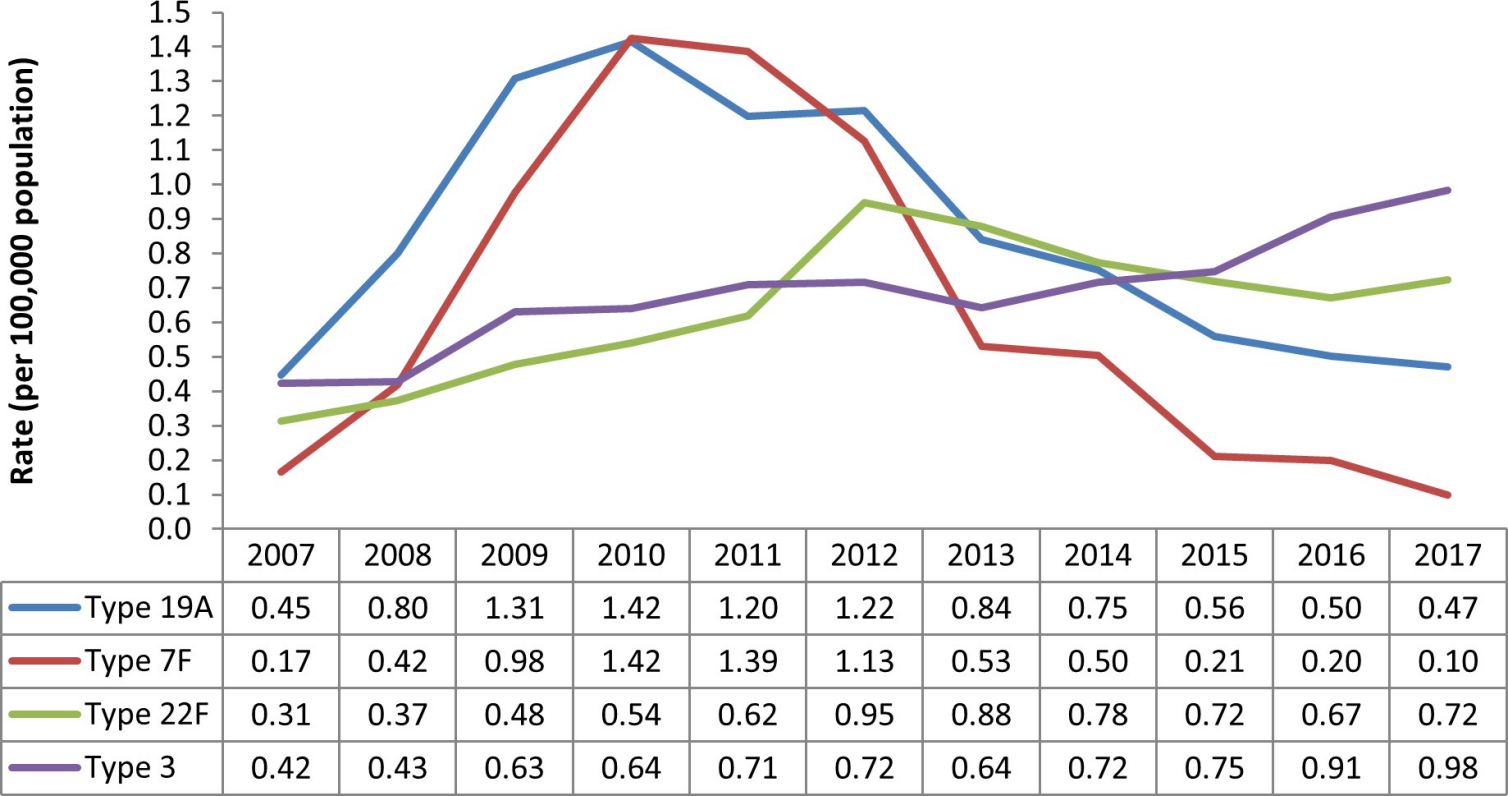
Incidence rates (per 100,000 population) of commonly^a^ reported *S*. *pneumoniae* serotypes in Ontario, Canada, by year: 2007–2017. ^a^ Serotypes 19A, 3, 22F and 7F individually accounted for ≥9.2% of cases with available serotype information (42.6% combined); other reported serotypes individually accounted for ≤ 4.2% of cases with available serotype information during the eleven year period, and were excluded.

In all, 70 different serotypes were reported during the study period. Of the 9,725 cases with available serotype information, the most commonly identified serotypes were 19A (13.1%), 3 (10.5%), 22F (9.8%) and 7F (9.2%), all four of which are vaccine-preventable and accounted for 42.6% of these cases; other reported serotypes individually accounted for less than 4.2% of cases with available serotype information during the entire eleven year period (**Fig 3**). Serotype 19A disproportionately affected younger populations, accounting for 26.6% of cases under five years with available serotype information (214/806). Annual incidence due to serotype 19A peaked in 2010 (at 1.42 per 100,000 population) and declined to 0.47 per 100,000 population in 2017 (*p* < .001), representing a 66.9% reduction in eight years. We observed a similar pattern for incidence due to serotype 7F, which decreased from 1.42 to 0.10 per 100,000 population between 2010 and 2017, a 93.0% reduction in eight years (*p* < .001). We also noted increasing trends in incidence due to both serotype 3 and 22F between 2007 and 2017; incidence due to serotype 3 increased from 0.42 to 0.98 per 100,000 population (*p* < .001), and incidence due to serotype 22F increased from 0.31 to 0.72 per 100,000 population (*p* < .001) (**Fig 4**). Among non-vaccine-preventable serotypes, 23A was the most commonly identified serotype (4.0%) (**Fig 3**).

## Discussion

In Ontario, the overall incidence of IPD has remained unchanged during the last eleven years despite comprehensive publicly-funded pneumococcal immunization programs. The decline in incidence due to PCV13 serotypes is offset by the rising incidence due to serotypes unique to PPV23 and non-vaccine-preventable serotypes, particularly in older age groups. The continued severity of IPD in Ontario is evident in measures of morbidity and mortality, with over 70% of cases requiring hospitalization and a case fatality ratio of more than 10% among adults 50 years and older. Our analysis also highlights the disproportionately higher burden of disease in northern Ontario (where many indigenous communities are located), aligning with previous findings of increased incidence in northern populations and among indigenous populations [17,18].

As illustrated in our analysis, the direct effect of Ontario’s pediatric PCV13 vaccine program, and the indirect effect of herd immunity, is clear in the PCV13-specific trends. The incidence of IPD due to serotypes covered by the PCV13 vaccine was rising rapidly in Ontario prior to PCV13 program implementation in 2010, notwithstanding the implementation of a universal PCV7 program in 2005; previous studies in Ontario also observed the high burden of serotypes 19A and 7F in the province during this pre-PCV13 program period [9,13]. Since implementation, there have been significant declines in incidence due to PCV13 serotypes, particularly in young children and older adults. The observed reductions in incidence due to serotypes 19A and 7F since 2010 is particularly noteworthy. Similar reductions in IPD incidence due to PCV13 serotypes have been observed in both pediatric and non-pediatric populations in other jurisdictions following PCV13 implementation [19,20]. Studies have also demonstrated decreased pneumococcal nasopharyngeal colonization in children (and therefore reduced exposure of *S*. *pneumoniae* to adults) following the introduction of the PCV13 vaccine [19,21,22]. While the effects of Ontario’s pediatric PCV13 program are apparent, provincial coverage of PCV13 is suboptimal–provincial coverage assessments (2013–2017) have found PCV13 coverage of approximately 80% [23–25]. The recent increases in incidence due to PCV13 serotypes among children under five years observed in our analysis is also concerning. While improving pediatric PCV13 coverage is necessary to maximize program impact in all age groups, further examination of both vaccine coverage and the distribution of specific serotypes among children under five years is needed to understand these recent increases.

In contrast to evidence that the PCV13 vaccine has impacted the epidemiology of most PCV13 serotypes, it appears to have had no impact on incidence due to serotype 3, which continues to rise. This trend has also been observed in several other jurisdictions [26–29] and is supported by evidence of lower efficacy of PCV13 against serotype 3 compared to other serotypes [21,29,30]. Further study is needed to examine the epidemiology of serotype 3 in Ontario and the effectiveness of PCV13 against this serotype. This would include further examination of serotype 3 trends by age, by vaccine program eligibility, and by individual level vaccination status.

Incidence due to serotypes unique to PPV23 and non-vaccine-preventable serotypes has risen continuously during the study period, across age groups but most noteworthy in older age groups. The increase in incidence due to serotypes unique to PPV23 may be due to poor vaccine coverage, lower vaccine effectiveness or vaccine failure. While Ontario-specific coverage estimates for adults are not available, the Public Health Agency of Canada has estimated that coverage for the PPV23 vaccine among Canadian adults 65 years and older was 41.6% in 2016, well below the national target of 80% [31]. Studies have also observed low effectiveness or only short-term protection of PPV23 against IPD in older adults [32–34]. The growing burden of serotype 22F (covered by PPV23 but not PCV13) that we observed has also been observed in other studies in Ontario and nationally [17,18,35,36]. While improving PPV23 coverage in older adults and other high risk populations could reduce the burden of PPV23 serotypes, the Ontario epidemiology suggests the need for a pneumococcal conjugate vaccine with broader serotype coverage [37]. Ongoing surveillance of circulating serotypes in the province should provide further evidence to inform Ontario’s pneumococcal vaccination program.

Despite the effectiveness of the PCV13 program in Ontario, our analysis shows high illness severity among cases, particularly among young children and older adults. Severity is particularly pronounced in the older age groups, with the majority of cases, hospitalizations and deaths occurring in those 50 years and older, as noted elsewhere [14,38,39]. While hospitalization among infants and young children in Ontario is high, case fatality is low compared to older ages. This might be explained by the important impact of pneumococcal conjugate vaccines on childhood deaths from IPD [40,41] and the availability of a publicly-funded pediatric PCV program in Ontario since 2005. Others have noted that older adults have a higher case fatality ratio compared to younger age groups in the conjugate vaccine era [39,42,43], as well as trends towards a higher case fatality in 50–64 year olds in the post-vaccine era than in the pre-vaccine era [2,44].

### Limitations

There are some limitations with this study. IPD cases are likely underreported in Ontario’s passive surveillance system due to variety of reasons, including health care seeking behaviours and variations in clinical testing. While the degree of underreporting in Ontario is unknown, it is expected that our data is likely skewed towards severe cases and the true burden is higher than we have estimated due to the nature of passive surveillance of invasive disease. On the other hand, the proportion of cases reported to have been hospitalized may be underestimated if hospitalization status was not available or reported to the public health unit. Small numbers, particularly in the infant age group, may cause unstable trends and should also be interpreted with caution. Certain variables are also underreported. Completeness of immunization history for cases in iPHIS is low, which provides challenges in interpreting trends, limits the ability to interpret vaccine effectiveness and limits comparisons of serotype-specific trends between vaccinated and unvaccinated populations, which is necessary to inform Ontario’s immunization program. In addition, as the change from PCV10 to PCV13 occurred concurrently with the schedule change from four to three doses, we are not able to determine the impact of either change independently. Risk factor information is also lacking, but is necessary to understand the impact of socio-demographic factors and comorbidities on IPD risk. Serotype data is also missing for 21.4% of cases, which limits generalizability of the observed trends. However, substantial improvements to completeness of serotype data were made over time during the study period (**S1 Fig**). Improving the collection of these variables would allow for a more thorough understanding of IPD and more informed public health action in Ontario.

## Conclusion

Eight years after PCV13 program implementation in Ontario, this analysis demonstrates the success of the PCV13 program in significantly reducing the serotype-specific burden of IPD. However, these reductions have been offset by increases in serotypes unique to PPV23 and non-vaccine-preventable serotypes, and, unfortunately, overall IPD incidence has not declined in the province. The rise of certain serotypes (namely 3 and 22F) and suboptimal vaccination coverage are potentially contributing factors. The shifting epidemiology and serotype distribution observed in this analysis highlights the importance of ongoing and robust surveillance, which can monitor circulating serotypes, detect serotype replacement, and inform Ontario’s pneumococcal immunization strategy.

## Supporting information

S1 Fig Number of confirmed invasive pneumococcal disease cases, by associated vaccine type^a^ and year, in Ontario, Canada, 2007–2017 (N = 12,377). ^a^ Cases with non-typeable serotypes were included in the non-vaccine-preventable category (n = 25). Abbreviations: PCV13: 13-valent pneumococcal conjugate vaccine; PPV23: 23-valent pneumococcal polysaccharide vaccine.
(TIF)
