# Supplementary figures and images for: The shifting epidemiology and serotype distribution of invasive pneumococcal disease in Ontario, Canada, 2007-2017
Source: PLoS One. 2019 Dec 13;14(12):e0226353. doi: 10.1371/journal.pone.0226353 (PMC6910703; doi:10.1371/journal.pone.0226353)

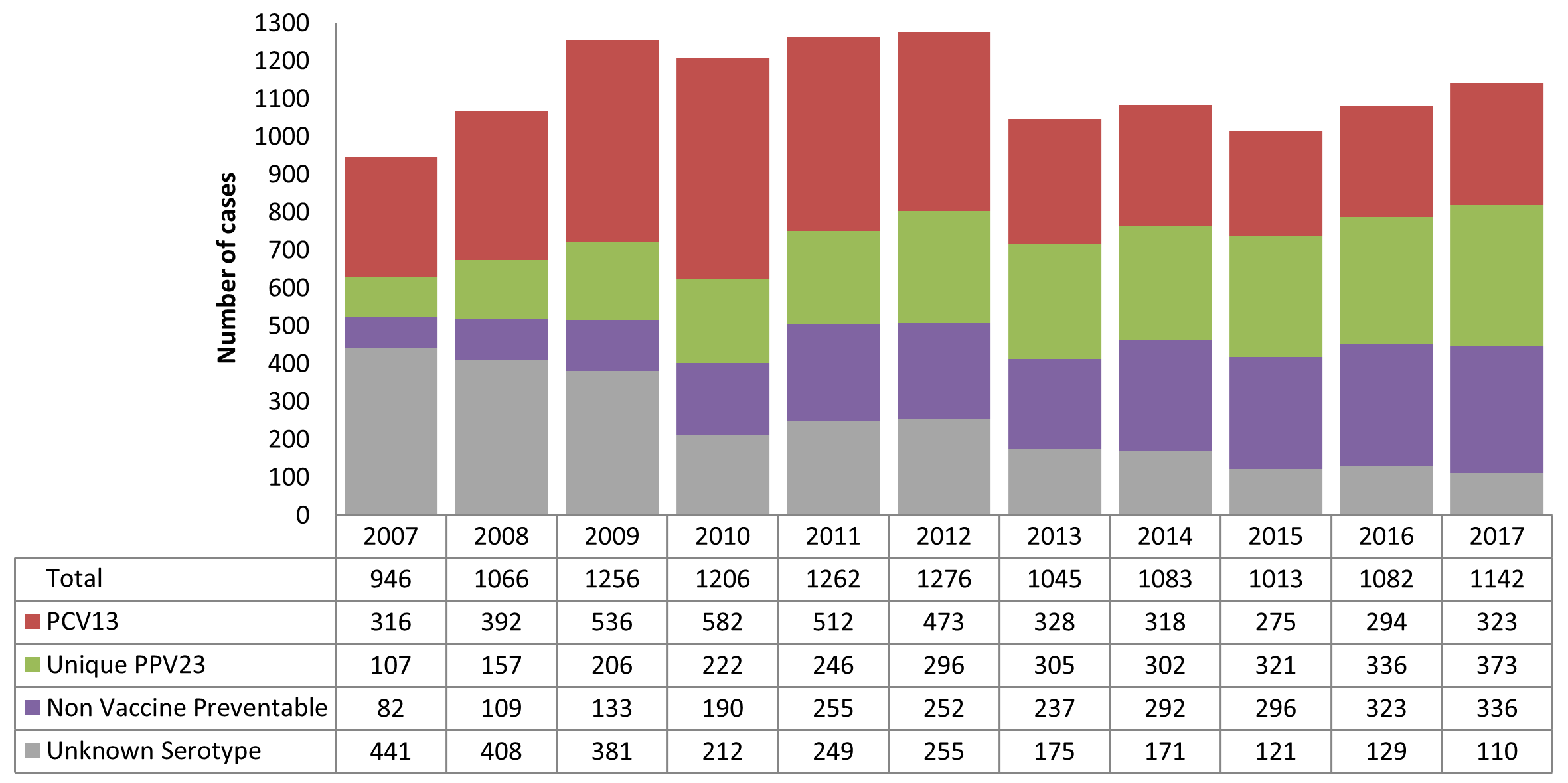

Supplement: S1 Fig — a Cases with non-typeable serotypes were included in the non-vaccine-preventable category (n = 25). Abbreviations: PCV13: 13-valent pneumococcal conjugate vaccine; PPV23: 23-valent pneumococcal polysaccharide vaccine. (TIF) [file pone.0226353.s001.tif]
